# A novel Ffu fusion system for secretory expression of heterologous proteins in *Escherichia coli*

**DOI:** 10.1186/s12934-017-0845-z

**Published:** 2017-12-21

**Authors:** Cheng Cheng, Shanshan Wu, Lupeng Cui, Yulu Wu, Tianyue Jiang, Bingfang He

**Affiliations:** 1 School of Biotechnology and Pharmaceutical Engineering, Nanjing Tech University, No. 30 South Puzhu Road, Nanjing, 211816 People’s Republic of China; 2 School of Pharmaceutical Sciences, Nanjing Tech University, No. 30 South Puzhu Road, Nanjing, 211816 People’s Republic of China; 3 Wuxi AppTec (Suzhou) Testing Technology Co.,Ltd., 1336 Wuzhong Avenue, Xinzhiyuan Building B, Wuzhong District, Suzhou, 215104 Jiangsu China

**Keywords:** Secretory expression, Fusion tag, *β*-fructofuranosidase truncations, Heterologous proteins, *Escherichia coli*

## Abstract

**Background:**

The high level of excretion and rapid folding ability of *β*-fructofuranosidase (*β*-FFase) in *Escherichia coli* has suggested that *β*-FFase from *Arthrobacter arilaitensis* NJEM01 can be developed as a fusion partner.

**Methods:**

Based on the modified Wilkinson and Harrison algorithm and the preliminary verification of the solubility-enhancing ability of *β*-FFase truncations, three *β*-FFase truncations (i.e., Ffu209, Ffu217, and Ffu312) with a native signal peptide were selected as novel Ffu fusion tags. Four difficult-to-express protein models; i.e., CARDS TX, VEGFR-2, RVs and Omp85 were used in the assessment of Ffu fusion tags.

**Results:**

The expression levels and solubility of each protein were markedly enhanced by the Ffu fusion system. Each protein had a favorable Ffu tag. The Ffu fusion tags performed preferably when compared with the well-known fusion tags MBP and NusA. Strikingly, it was confirmed that Ffu fusion proteins were secreted into the periplasm by the periplasmic analysis and N-amino acid sequence analysis. Further, efficient excretion of HV3 with defined anti-thrombin activity was obtained when it was fused with the Ffu312 tag. Moreover, HV3 remained soluble and demonstrated notable anti-thrombin activity after the removal of the Ffu312 tag by enterokinase.

**Conclusions:**

Observations from this work not only complements fusion technologies, but also develops a novel and effective secretory system to solve key issues that include inclusion bodies and degradation when expressing heterologous proteins in *E. coli*, especially for proteins that require disulfide bond formation, eukaryotic-secreted proteins, and membrane-associated proteins.

**Electronic supplementary material:**

The online version of this article (10.1186/s12934-017-0845-z) contains supplementary material, which is available to authorized users.

## Background

In recent research*, Escherichia coli* (*E. coli*) has established itself as one of the most extensively used systems in the industrial production of recombinant proteins, which owes much to the high yield of production that is possible with this system, the low manufacturing cost, a well-characterized expression system, and amenability to high cell-density fermentation biotechnology [1, 2]. In addition, due to a lack of appropriate folding-assistance proteins and cofactors that can mediate post-translational modifications, the likelihood of inclusion body formation is considerately high when heterologous proteins are expressed in *E. coli* [3]. However, continuous endeavors have been devoted to overcome such limitations. With the aid of genetic and protein engineering strategies, there are many solutions that can potentially provide successful expression of soluble recombinant proteins in an *E. coli* expression system, including choosing a suitable expression host and vector, codon usage, sequence optimization, inducer concentration, expression temperature, fusion technology and co-expression of a chaperone.

Therapeutic and diagnostic proteins as biopharmaceuticals have been applied widely particularly in respect of the broader interest in recombinant technology. These proteins are required in sufficient quantities; however, it remains a challenge to obtain enough proteins from natural sources. To express eukaryotic proteins in *E. coli*, the concept of fusion technology represents an effective method to enhance protein solubility [4]. In a contemporary study, fusion expression vectors provide useful tools to strengthen the solubility and productivity of eukaryotic proteins, including Maltose-binding protein (MBP), N-utilization substance A (NusA), Thioredoxin A (Trx) and translation initiation factor 2 domain I (IF2). Furthermore, it has recently been determined that fusion partners like the *Fasciola hepatica* 8 kDa antigen (Fh8), the *E. coli* secreted protein A (EspA) and KDPG aldolase (EDA) have emerged with the intent of improving synthetic yields, enhancing solubility and folding processes and simplifying the purification of eukaryotic proteins [3–5]. Related methods have also been used for the production of functional proteins; for example, the peripheral cannabinoid receptor was successfully expressed on using MBP, Trx, Sterp-tag and polyhistidine tags [6].

Fusion partners, like IF2 and small ubiquitin modifying protein (SUMO), have dramatically improved the soluble expression of recombinant Heparinase I [7]. The Fh8 fusion tag acts as an effective solubility enhancer that aims to improve the expression of six eukaryotic proteins from different organisms that include *Artocarpus incisa*, *Cryptosporidium parvum* and *Saccharomyces cerevisiae*. Moreover, high soluble expression of these proteins was achieved with the aid of the Fh8 tag [8]. Although commonly used fusion tags are effective in large part by solving inclusion bodies and improving expression levels, there is currently no guarantee of success. Determining the correct fusion partners to optimize heterologous protein expression is not empirical but largely depends on the passenger protein. Fusion partners are protein specific and not a universal method for solubilizing passenger proteins [4]. Current fusion expression partners still need to be improved with the aim of properly expressing various heterologous proteins. Thus, the development of novel fusion tags that permit effective expression and enhanced solubility of heterologous proteins is an urgent need.

In this study, five heterologous proteins, which represent key proteins of pathogenic infectious microorganisms and targets for therapeutic vaccines, were used to evaluate the effectiveness of the Ffu fusion system. Specifically, we used the *Mycoplasma Pneumonia* community-acquired respiratory distress syndrome toxin (CARDS TX), vascular endothelial growth factor receptors-2 (VEGFR-2), rubella virus structural polyproteins (RVs), *Chlamydia major* outer membrane protein (Omp85), and hirudin variant III (HV3).

In this system, CARDS TX that was derived from *Mycoplasma pneumoniae* served as an exotoxin, which is translocated for its immune dominance to be apparent [9]. Furthermore, therapeutic inhibition of VEGFR-2 plays an important role in the clinical therapy of several diseases including tumor angiogenesis [10, 11]. Rubella virus structural proteins are synthesized as a polyprotein precursor in association with the endoplasmic reticulum of the host cell [12]. In addition, the Omp85 family of proteins are necessary for outer membrane biogenesis in mitochondria and bacteria [13]. HV3 is applicable in the setting of treating cataract and in prophylaxis [14].

The CARDS TX and HV3 are integral components of native secretory proteins. Moreover, VEGFR-2, RVs and Omp85 belong to the broad family of membrane-associated proteins, and their physiological features make their production in *E. coli* very challenging. Disulfide bridges that are present in the secreted proteins like CARDS TX, RVs and HV3 [15–17], can stabilize protein structures. For example, Cys–Cys bridges block folding units into a stable conformation by linking residues in a covalent manner, which is necessary for the correct folding and stability of the tertiary structure of these proteins. The most direct method to exploit *E. coli* for recovering folded disulfide bond-dependent proteins is to instruct the migration of the translated polypeptides to the bacterial periplasm [18]. The greater oxidative environment of the periplasmic space serves to facilitate the correct structural organization of disulfide bonds than would otherwise be seen in the cytoplasm [19, 20]. Further, there is comparatively decreased protease activity and fewer contaminating proteins in the periplasm than are commonly found in the cytoplasm. This is beneficial if one wishes to avoid protease attack and to simplify protein purification. Successful secretion of these proteins into the periplasm or their excretion into the culture medium is more beneficial as compared with their intracellular expression. However, the secretory ability of *E. coli* is often limited to a discouragingly low level as compared with other hosts. This is due in part to its complicated bi-layered cellular envelope, and the production of heterologous proteins in the periplasm, which is often reported to be low [21, 22]. In addition, a limited number of fusion tags are available that can improve protein accumulation in the periplasm [18, 23].

In this current study, we found that in *E. coli*, *β*-FFase could be excreted into the medium in large quantities when expressed with its native signal peptide from *Arthrobacter arilaitensis* NJEM01, and does so over a comparatively short period of time. This means that the proteins are folded quickly with an efficient excretion pathway. Three different truncated lengths of *β*-FFase (i.e., Ffu209, Ffu217, and Ffu312) of 209, 217, and 312 residues in size, with a native signal peptide were selected and designed as Ffu fusion system, which compared favorably with the well-known fusion tags, MBP and NusA [24]. Interestingly, by using the Ffu fusion system, CARDS TX, VEGFR-2, RVs and Omp85 not only were successfully expressed in the soluble form, but were also secreted in the periplasmic space in *E. coli*. Additionally, HV3 achieved extracellular expression when exhibiting the Ffu312 fusion.

## Results

### Efficient excretory expression of *β*-FFase in *E. coli* and solubility prediction

In our previous research [25], *β*-FFase was isolated from *Arthrobacter arilaitensis* NJEM01 and was commonly used as a biocatalyst in sucrose hydrolysis and transfructosylation reactions, a process that permits greater amenability of glycoside synthesis in natural products. In addition, efficient soluble expression of the mature *β*-FFase in the cytoplasm was achieved in *E. coli*.

**Fig. 1:**
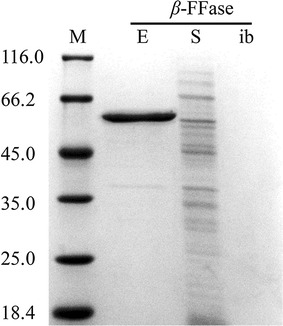
Excretory expression of *β*-FFase induced with 1 mM IPTG at 30 °C. Key: protein marker (M); excretion to the culture medium (E); soluble fractions (S); inclusion bodies (ib)

In the present study, and based on bioinformatic analyses, *β*-FFase consisted of a mature *β*-FFase comprising 495 residues and a signal peptide of 53 residues. It was found that the native signal peptide of *β*-FFase could translocate significant amounts of *β*-FFase to the culture medium in as little as 6 hours (Fig. 1). *β*-FFases with their native signal peptides are highly secretory proteins, this feature of *β*-FFase, has stimulated interest in developing novel fusion tags.

**Fig. 2:**
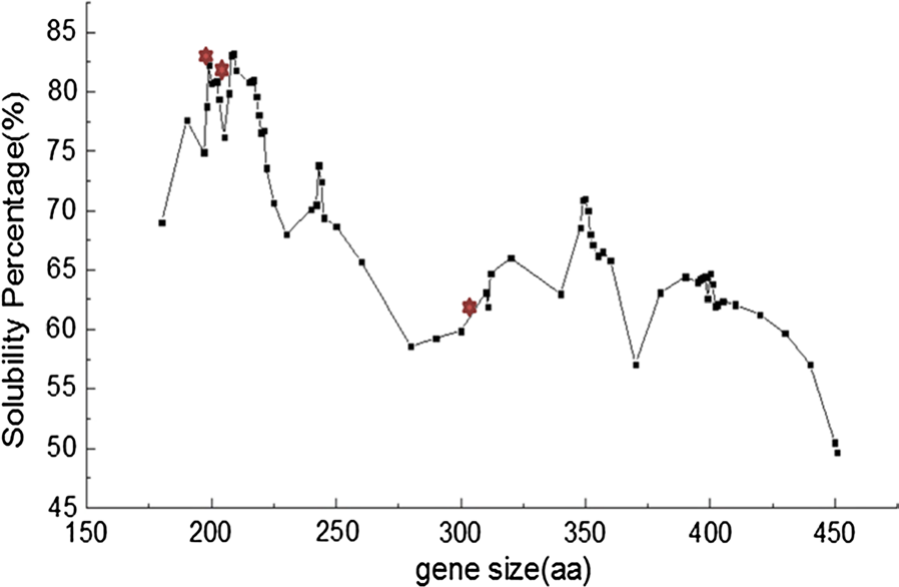
The predicted solubility of *β*-FFase truncations by the modified Wilkson and Harrison model. The selected *β*-FFase truncations (i.e., Ffu209, Ffu217 and Ffu312) were marked with a red hexagon

The predicted solubility of the *β*-FFase truncations and the display of different residue lengths by the Wilkson and Harrison model are shown below (Fig. 2). Based on the atom economy and preliminary verification of solubility-enhancing capability, the N terminal *β*-FFase truncations, which are referred to as Ffu209, Ffu217 and the Ffu312 tag with respective molecular weights of 17.7, 18.9 and 29.5 kDa after cleaving the native signal peptide were selected. The grand average values of hydropathicity (GRAVY) show the predominantly hydrophilic nature of Ffu209, Ffu217 and the Ffu312 tag. The features of the pFfu209, pFfu217 and pFfu312 vectors are also shown below (Table 1). The moderately small sizes and highly excretory ability of the Ffu209, Ffu217 and Ffu312 tags make them promising fusion tags. It was reported that MBP and NusA were ranked as two of the most popular tags for improving the solubility of their passenger proteins [8, 26–28]. Thus, they were used to compare the performance of the Ffu fusion tags.

**Table 1:** Features and properties of Ffu fusion tags used in this work

Fusion tags	Predicted protein solubility (%)	Tag size with signal peptide (aa)	Tag size without signal peptide (aa)	p*I*	MW (*kDa*)	GRAVY	Cys (%)	Promoter	Selection	Protease cleavage site
Ffu209	83.2	209	156	4.71	17.6	− 0.937	0	T7/lac	kan	EK
Ffu217	81	217	164	4.77	18.8	− 0.955	0	T7/lac	kan	EK
Ffu312	71	312	259	5.04	29.5	− 0.894	0	T7/lac	kan	EK

*aa* amino acids, *pI* isoelectric point, *MW* molecular weight, *Da* dalton

### Expression of target proteins (CARDS TX, VEGFR-2, RVs, and Omp85) fused with Ffu tags, MBP and NusA

**Fig. 3:**
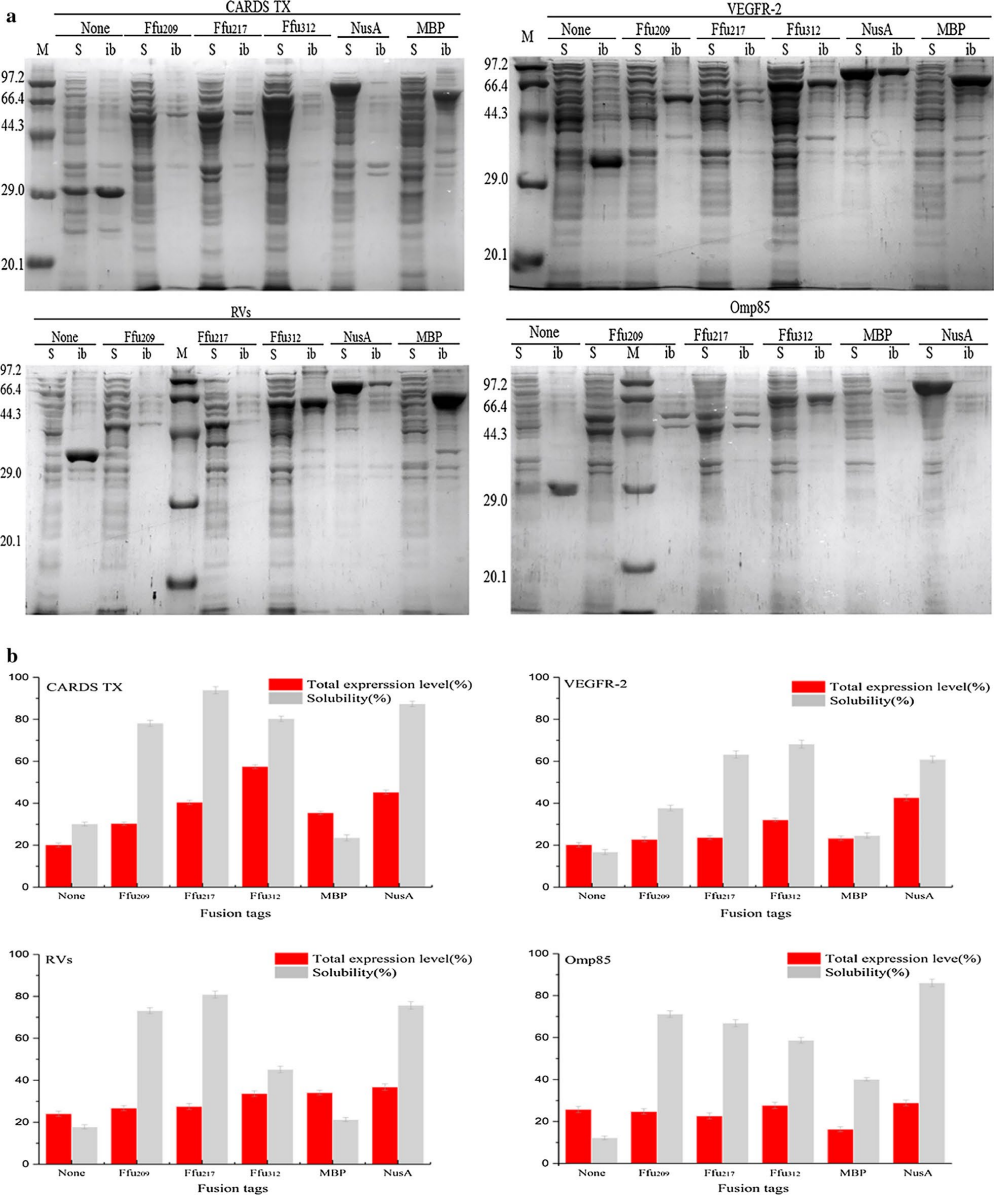
**a** Expression of targeted proteins (i.e., CARDS TX, VEGFR-2, RVs, and Omp85) with the fusion tags Ffu209, Ffu217, Ffu312, MBP and NusA. The expression condition was 0.5 mM IPTG at 25 °C. Key: soluble fractions (S); inclusion bodies (ib). **b** Analysis of the expression of the Ffu fusion as compared direct expression, MBP fusions and NusA fusions. Total expression levels and solubility of the targeted proteins were calculated by Quantity One version 4.62 (Bio-Rad, USA) software, and was done three times

The selected proteins CARDS TX, VEGFR-2, RVs and Omp85 are derived from different organisms and have different locations, functions and applications. GRAVY analysis (Table 2) presented comparatively high hydrophobicity of these target proteins. Their physiological features mean that soluble production in *E. coli* is very challenging. As shown in Fig. 3, the solubility of VEGFR-2, RVs and Omp85 were extremely low (i.e., less than 18%) when directly expressed in *E. coli,* with the exception that CARDS TX achieved an approximate 30% soluble expression. Efficient soluble protein expression was achieved with the aid of the Ffu fusion tags. Moreover, both the expression levels and solubility of the target proteins were improved to varying degrees as compared with more conventional direct expression approaches. Each protein (i.e., CARDS TX, VEGFR-2, RVs and Omp85) reacts differently to the presence of different solubility-enhancing tags. The target proteins CARDS TX and RVs favor the Ffu217 tag. VEGFR-2 favors the Ffu312 tag, and Omp85 optimally fits the Ffu209 tag. Specifically, the solubility of CARDS TX (28 kDa) was significantly improved by the Ffu fusion tags from 30.1% [pET28a (+)] to 94% (pFfu217), and the Ffu209 fusions of CARDS TX resulted in similar levels of soluble protein as was seen with the Ffu312 fusions, and displayed 78.08 and 80.23% solubility respectively. With regard VEGFR-2 (30.8 kDa), the Ffu312-VEGFR-2 fusion performed better than did the Ffu209-VEGFR-2 or Ffu217-VEGFR-2 fusions, wherein the solubility of Ffu312-VEGFR-2 was approximately 68.19%, which represented a more than fourfold higher level of expression than was seen with direct expression. For RVs (35.2 kDa), the Ffu217-RVs presented with higher soluble levels (80.86%) of protein than was seen with Ffu209-RVs or Ffu312-RVs. The proportion of soluble protein of Omp85 (27.8 kDa) was improved significantly under conditions where it was fused to the Ffu fusion system, and Ffu209-Omp85 showed the highest level of solubility (71.2%).

**Table 2:** Features and properties of target genes used in this study

Synthetic genes	Genbank/unioprot	Organism	Gene size (aa)	MW (kDa)	Cys (%)	GRAVY
*Mycoplasma pneumonia* community acquired respiratory distress syndrome toxin(CARDS TX)	AP017319.1	*Mycoplasma pneumoniae*	248	28.0	0.8	− 0.461
Vascular endothelial growth factors and receptors2 (VEGFR2)	EU826563.1	Homo sapiens	274	30.8	3.3	− 0.257
Rubella virus structural polyprotein (RVs)	AB706305.1	Rubella virus	319	35.2	3.8	− 0.382
Outer membrane protein (*Omp85*)	LN847043.1	*Chlamydia pneumoniae*	253	27.8	0.0	− 0.397
Hirudin variant III (*HV3*)	KX215716.1	*Hirudo orientalis*	67	7.17	9.0	− 0.848

The expression of target proteins using MBP and NusA fusion tags was also presented in Fig. 3. For the selected proteins, the Ffu fusion system performed better than did MBP in terms of both the expression level and solubility fraction. In the case of Omp85, though MBP-Omp85 presented the highest solubility among the MBP fusions, both the expression level and solubility of MBP-Omp85 remained lower than was found for Ffu-Omp85. In addition, although NusA tag behaved well in improving the expression level and solubility of the target proteins, Ffu fusion tags were also highly effective at enhancing protein solubility. Mostly, the Ffu fusion proteins presented similar solubility as did NusA fusions in the case of CARDS TX, VEGFR-2 and RVs (Fig. 3). Specifically, the fusion protein CARDS TX that was fused to Ffu217 presented similar solubility (94%) as was seen with Nusa-CARDS TX (87%). More soluble levels of VEGFR-2 fused with Ffu217 and Ffu312 were detected as compared with those counterparts that had been fused with NusA. Similarly, the soluble proportion of Ffu209-RVs and Ffu217-RVs were somewhat higher than was found for NusA-RVs. NusA-Omp85 presented with higher degrees of solubility and yield (86 and 28.9%) than was seen with Ffu209-Omp85 (71.2 and 24.8%). However, larger fusion partners could result in an over-optimistic evaluation of protein solubility and yield [3, 29]. The molecular weight of Ffu209 based on the atom economy, was only one-third that of NusA. Thus, Ffu209 also performed well in terms of the enhanced expression and solubility of Omp85 as compared with NusA.

Moreover, when taking into account the possibility of enhanced solubility influencing His-tag to target proteins, we tested the expression levels of target protein fusions that had been fused to His tag (please also refer to Additional file 1: Figure S1). No yield improvement was seen for soluble proteins when it was expressed with His tag—an observation that was consistent with previously published results [29, 30].

### Secretory expression of target proteins

**Fig. 4:**
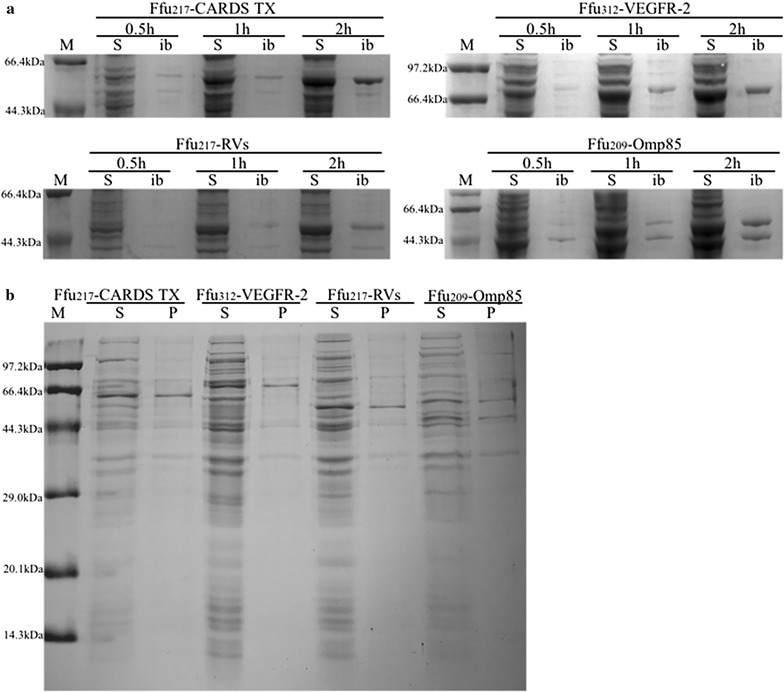
**a** Secretory expression of Ffu-fused target proteins in *E. coli* at time intervals of 0.5, 1, and 2 h. The conditions for expression of the proteins were: 0.5 mM IPTG at 25 °C. Key: soluble fractions (S) and inclusion bodies (ib). **b** The periplasmic analysis of Ffu-fused targeted proteins in *E. coli*. The conditions for expression of the proteins were: 0.5 mM IPTG at 25 °C for 1 h. Key: soluble fractions (S) and periplasmic fractions (P)

Strikingly, the efficient secretion of all target proteins (i.e., CARDS TX, VEGFR-2, RVs and Omp85) was achieved when using Ffu fusion tags. In this process, the most suitable Ffu tag for each target protein was selected for analysis of the expression of these fusion proteins, namely Ffu217-CARDS TX, Ffu312-VEGFR-2, Ffu217-RVs, and Ffu209-Omp85. The expression of Ffu-fused target proteins in *E. coli* were analyzed at time intervals of 0.5, 1, and 2 h. The fusion proteins progressively accumulated in the soluble fractions as the culture grew (Fig. 4a). Additionally, SDS-PAGE analysis of purified fusion protein Ffu217-CARDS TX following osmotic shock was shown in Additional file 1: Figure S2. Analysis of the N-terminal sequence of purified fusion protein Ffu217-CARDS TX showed that it was N-ATEPVPGF- (Fig. S3), which was completely consistent with the mature N-terminal sequence of *β*-FFase after removing the signal peptide [25]. This result, combined with periplasmic analysis (Fig. 4b), suggested that Ffu fusion proteins were translocated to the periplasmic space with the cleavage of signal peptide.

### Extracellular expression of Ffu312-HV3, biological activities of Ffu312-HV3 and HV3

**Fig. 5:**
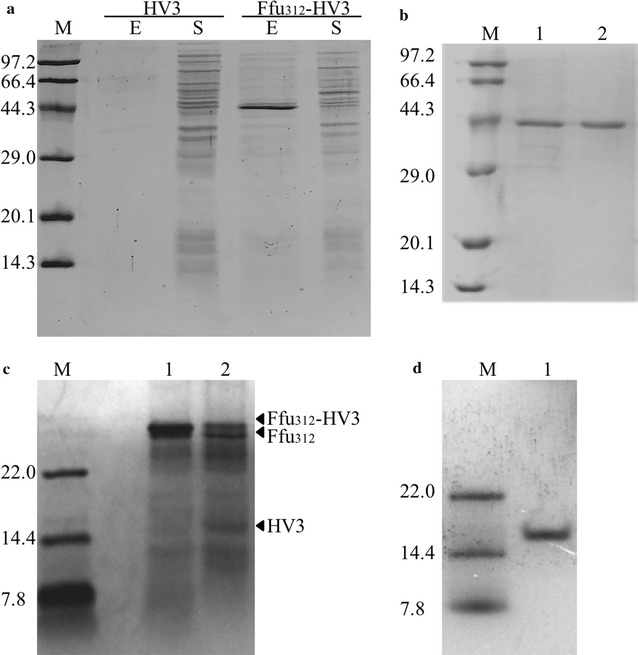
**a** Comparative expression of the target protein HV3. Key: expression of HV3 without any fusion tag (HV3); expression of HV3 with the Ffu312 tag (Ffu312-HV3); excretion to the culture medium (E); soluble fractions (S). **b** Purification of the fusion protein Ffu312-HV3. Key: supernatant of the fermentation broth (Lane 1); the purified protein Ffu312-HV3 (Lane 2). **c** SDS-PAGE analysis of fusion protein Ffu312-HV3 digested with enterokinase. Key: fusion protein Ffu312-HV3(Lane 1); fusion protein Ffu312-HV3 digested by EK (Lane 2). **d** Tricine/SDS-PAGE analysis of purified HV3. Key: purified HV3 (Lane 1)

Another possible consequence for heterologous expression, with the notable exception of the formation of inclusion bodies, results from the failure to rapidly reach a native conformation or to interact with folding modulators in a timely fashion—a process that is known as degradation [1]. HV3 with three intramolecular disulfide bonds is a secreted small polypeptide. It has been proven that cytoplasmic expression in *E. coli* is difficult for those small eukaryotic polypeptides that contain intramolecular disulfide bonds, due to the difficulty in correctly forming disulfide bridges in the cytoplasm and the degradation caused by various proteases [31, 32]. Thus, the Ffu fusion tags were expected to protect HV3 from degradation by promoting the translocation of HV3. Previous research showed that the Ffu312 fusion tag was a favorable Ffu fusion tag for HV3. As Fig. 5a demonstrated, HV3 was not expressed in pET-28a (+), which might result from proteolytic degradation. However, HV3 was expressed at high levels when fused to the Ffu312 tag, and significant levels of the fusion protein Ffu312-HV3 were excreted into the culture medium.

Next, Ffu312-HV3 was highly purified from the clarified supernatant by nickel affinity chromatography. SDS-PAGE analysis of the purified fusion protein Ffu312-HV3 demonstrated that the purity of the fusion protein was almost 90% (Fig. 5b). Approximately 800 mg/L of the fusion protein Ffu312-HV3 could be obtained. As observed in Fig. 5c, tricine/SDS-PAGE analysis demonstrated that HV3 was released after incubation of the fusion protein Ffu312-HV3 with EK. Nickel affinity chromatography was used to obtain the cleaved HV3 product after the thorough digestion. Approximately 132 mg of HV3 was obtained from a 1 L culture of *E. coli* BL21 (DE3) cells harboring the pFfu312-HV3 plasmid. As shown in Fig. 5d, tricine/SDS-PAGE analysis showed that the mass value of HV3 was approximately 15 kDa, which implied that the excreted HV3 formed a dimer during expression. It was speculated that the strong hydrophobic interaction between two hirudin molecules might have resulted in dimer formation. This phenomenon was also observed in previously published literature [17, 33].

The anti-thrombin activity analysis was used to assess the biological activity of the purified Ffu312-HV3 and HV3. The activity of purified Ffu312-HV3 reached 2625 ATU/mg. After the cleavage of the Ffu312 tag, the anti-thrombin activity of the fusion-free HV3 was significantly improved to 10,906 ATU/mg, which is more than four times that of Ffu312-HV3. In this study, the anti-thrombin activity of purified HV3 almost achieved the same levels as those previously published [17]. The high level of solubility and anti-thrombin activity of purified Ffu312-HV3 and HV3 reflected the native conformation of HV3.

## Discussion

Fusion tags are employed to solve key problems including inclusion bodies, low expression levels and sensitivity to proteolytic degradation. However, no universal tags exist for soluble expression of heterologous proteins [34]. In this current work, the novel Ffu fusion tags were presented to enrich the fusion tag pool and to provide more options for efficiently improving the soluble and secretory expression of heterologous proteins in *E. coli*.

The utility of the Ffu tags was strongly demonstrated by the drastic improvement in the solubility and total yield of heterologous proteins (CARDS TX, VEGFR2, RVs and Omp85) in *E. coli*. For these selected proteins, their direct expression in *E. coli* led to a significant proportion of inclusion bodies. However, CARDS TX, VEGFR-2, RVs and Omp85 achieved higher yields and solubility when fusing to Ffu fusion tags. Besides, each of these heterologous proteins has individually applicable Ffu fusion tags.

In the present study, MBP fusions that were expressed into a mass of inclusion bodies, presented a slight advantage when compared with direct expression of the studied proteins. Though NusA fusions presented with high solubility and expression levels, the Ffu fusions also presented remarkable solubility and yield. In addition, the smaller size of Ffu209, Ffu217 and Ffu312 tags (17.7, 18.9 and 29.5 kDa) were more advantageous than MBP (40 kDa) and NusA (54.8 kDa). Larger fusion partners might contribute to an over-estimation of the solubility and yield [8, 26, 29]. Besides, larger fusion partners may increase the energy required to obtain the requisite numbers of molecules and result in larger steric hindrance [34]. Thus, the Ffu fusion tags with smaller sizes display a more preferable performance when compared with the commonly-used tags MBP and NusA in enhancing the solubility and improving the yields of heterologous proteins. This result also confirmed that although the MBP and NusA fusion expression systems are well-known fusion tags for enhancing the solubility of passenger proteins, none of these tags work universally well with every passenger protein, and thus alternatives are needed.

In the past few decades, only limited numbers of fusion tags, including MBP that can translocate target proteins to the periplasm [35], were discovered for enhancing the translocation and solubility of heterologous proteins [36]. In this study, MBP failed to translocate the studied proteins to the periplasm. MBP fusions aggregated into the cytoplasm and led to the formation of a mass of inclusion bodies. However, the Ffu fusion tags with a native signal peptide successfully translocated significant amounts of target proteins (CARDS TX, VEGFR-2, RVs and Omp85) into the periplasm of *E. coli*. Promoting the translocation of the target proteins to the periplasm where it is known that lower levels of proteases exist, not only can protect target proteins from degradation [37], but also contribute to the disulfide bonds formation and subsequent correct folding of the target proteins [34].

As for small eukaryotic polypeptides that contain intramolecular disulfide bonds, such as HV3, the degradation caused by various protease and their difficulty in forming disulfide bridges in the cytoplasm correctly makes their expression in *E. coli* difficult [32]. The secretion process benefits the stability and correct folding of such eukaryotic proteins. Strikingly, the efficient excretion of HV3 was obtained when it was fused to the Ffu312 tag, while almost no expression was detected when it was directly expressed following its cloning in pET28a (+) and *E. coli*.

It is noticed that there are many bands in the excreted fraction in Fig. 5a, this might because the metabolic stress in bacterial generating recombinant proteins such as secreted proteins can cause cell lysis during the latter fermentation [38]. The anti-thrombin activity of HV3 was used to illustrate the difference of target proteins before and after the removal of the Ffu fusion system. The high anti-thrombin activity of Ffu312-HV3 and HV3 showed that HV3 retains its native structure. While its activity has been improved significantly after the removal of the Ffu312 tag, this is likely because the steric hindrance imposed by the fusion partner Ffu312 on the passenger protein HV3 was eliminated. A question that one should ask is why HV3 is *excreted* and the remaining proteins are *secreted*? One reason that could explain this phenomenon is that the effect of enhancing the solubility of heterologous protein is protein specific. The secretion/excretion efficiency depends largely on the properties of the target proteins [1, 3]. Moreover, the character of HV3, which includes its small size might allow it to translocate more easily to the culture medium with the aid of the Ffu tags.

Though several hypotheses have been suggested to explain the mechanisms by which fusion tags improve the solubility of passenger proteins, the exact mechanism of this remains unclear [5]. Further study is still underway to confirm which hypothesis best fits the observed mechanism of the Ffu fusion tags; however, it is certain that the native signal peptide of *β*-FFase plays an important role in the efficient translocation of the passenger proteins to the periplasmic or even to the culture medium. Additionally, the high solubility and stability of the Ffu fusion tags are also a prerequisite for the secretory of passenger proteins.

## Conclusions

*E. coli* is widely used for the production of therapeutic proteins. However, it remains challenging to increase yields and facilitate the correct folding of heterologous proteins, especially those containing disulfide bonds for diagnostic, therapeutic and other purposes. In this study, the Ffu fusion tags provided an effective approach for the soluble production of target proteins, and offer several advantages in improving the secretory and extracellular expression of passenger proteins in *E. coli*. Furthermore, the Ffu fusion tags worked better than did both of the well-acknowledged fusion tags (i.e., MBP and NusA) in expressing the four targeted proteins. Besides, the Ffu312 tag renders significant excretion of HV3 and helps it retain its native structure both before and after the removal of the Ffu tags. We envision that these Ffu fusion tags have the potential of wide applicability in producing other important disulfide bond-dependent proteins, native secretory proteins and membrane-associated proteins in the periplasm or in the culture medium of an *E. coli* expression system.

## Methods

### General

The *E. coli* DH5a (Invitrogen) was used for sub-cloning and plasmid amplification, and the *E. coli* BL21 (DE3) (Novagen) was used for protein expression. The pET-28a (+) (Novagen) was used as an expression backbone, the restriction enzymes *Nco*I, *Xho*I and *Nde*I were purchased from Takara Bio. All the DNA ligation reactions were carried out with T4 DNA ligase (TakaRa). Low molecular weight protein marker was purchased from Beijing Solarbio Science & Technology Co. Ltd.

### Solubility prediction of *β*-FFase truncations and construction of the Ffu_209_, Ffu217 and Ffu312 vectors

The modified Wilkinson and Harrison algorithm was used to predict the solubility of *β*-FFase truncations. The *β*-FFase truncations with higher predicted solubility values were selected to construct the pET28a (+) expression vector, following which, the expression level and solubility-enhancing ability of *β*-FFase truncations were evaluated. Based on the atom economy principle, the *β*-FFase truncations (i.e., Ffu209, Ffu217, Ffu312) with 209, 217 and 312 residues with a signal peptide were chosen in this study. The enterokinase (EK) sequence was used in this study to remove the fusion tags (Ffu209, Ffu217, and Ffu312). His tag sequence was placed at the C-terminal domain of target proteins.

The construction of p*β*-FFase, pFfu209, pFfu217 and pFfu312 vectors were manipulated as described here: four sets of the primers F1&R2, F1&R3, F1&R4 and F1&R5 (Additional file 1: Table S1) were used to amplify the *bff, ffu209, ffu217* and *ffu312* fragments from the genomic DNA of strain NJEM01. Then purified PCR products and the pET28a (+) plasmid were digested with *Nco*I and *Nde*I restriction enzymes, final p*β*-FFase, pFfu209, pFfu217, pFfu312 vectors were obtained after the ligation of the digested DNAs by T4 DNA ligase. Recombinant plasmids were transformed into *E. coli* BL21 (DE3). The resulting clones harboring recombinant plasmids of p*β*-FFase, pFfu209, pFfu217, pFfu312 vectors were confirmed by sequencing. Cells containing these vectors were grown to mid-log phase (OD_600_ = 0.6) at 37 °C in LB-broth with 50 µg/L kanamycin. Over-production of fusion protein was induced with IPTG at a final concentration of 1 mM at 30 °C. Then cells were harvested when the OD_600_ of the culture reached 3.0, the supernatants of the cultures (excretion) were collected by centrifugation, and pelleted cells were resuspended with 20 mM Tris–HCl buffer (pH 6.5) and lysed by ultrasonication, the supernatants of total lysate were obtained by centrifugation. Finally, 12% SDS-PAGE was used to analyze the supernatant of the culture medium (excretion) and the supernatant of total lysate (soluble fractions).

### Cloning and expression of the target proteins (CARDS TX, VEGFR-2, RVs and Omp85) with the Ffu, MBP, and NusA fusion tags

The pMBP and pNusA vectors were constructed using the same method as that described for pFfu209, pFfu217, and pFfu312 vectors. The primers with restriction sites *Nde*I and *Xho*I were used to amplify the target genes (*cards tx*, *vegfr*-*2*, *rvs*, *omp85* and *hv3*) and are shown in the Additional file 1: Table S1. After ligation of the digested PCR products and plasmids, these target genes were inserted into the constructed vectors (i.e., pFfu209, pFfu217, pFfu312, pMBP and pNusA), then the constructions were transformed into *E. coli* BL21 (DE3). Measurements of protein expression and solubility were performed essentially as was described above, except with 0.5 mM IPTG induced at 25 °C. The protein concentrations were determined by the Bradford method using bovine serum albumin as the standard. The periplasmic analysis was performed by the method of TSE extraction procedure, which uses a Tris-sucrose solution supplemented with EDTA as described previously [39].

### N-terminal amino acid sequence analysis

Protein disulfide bonds of the samples of target protein were reduced for 40 min with 5 mM dithiothreitol at room temperature and alkylated for 40 min with 15 mM iodoacetamide in the dark. The alkylated protein samples were digested overnight at 37 °C with trypsin (Promega, V5113) or chymotrypsin (Promega, V1061) in a 1:50 enzyme-to-substrate ratio. Following the digestion, the peptide mixtures were acidified with trifluoroacetic acid (TFA) to 1%, and desalted by home-made C18 tip. The desalted peptide samples were dried in a vacuum concentrator and produce purified peptide samples. The dried peptides were dissolved in 10 μL of 0.1% formic acid in water and subjected to nanoLC-MS/MS analysis. The raw MS files were analyzed and searched against protein sequence database using Proteome Discoverer. The parameters were set as follows: the protein modifications were carbamidomethylation (C) (fixed), oxidation (M); the enzyme specificity was set to trypsin; the maximum missed cleavages were set to 2; the precursor ion mass tolerance was set to 10 ppm, and MS/MS tolerance was 0.6 Da. Only high confident identified peptides were chosen for downstream protein N-terminal amino sequence analysis.

### Purification and biological activities of the fusion proteins Ffu312-HV3 and HV3

The fusion protein Ffu312-HV3 was purified from the clarified supernatant after centrifugation and ultrafiltration. Thereafter, nickel affinity chromatography was used to purify Ffu312-HV3, which was done using a 1-mL NTA-Ni^2+^ agarose column (QIAGEN) that was equilibrated with the binding buffer (50 mM Tris–HCl, 0.5 M NaCl pH 8.0), and the eluted solution was then applied to an equilibrated Ni^2+^-chelating column. Elution buffer (50 mM Tris–HCl, 0.5 M NaCl, 0.3 M imidazole pH 8.0) was used to elute the bound proteins at a 1 mL/min flow rate. Next, 12% SDS-PAGE was applied to analyze the expression and purification of Ffu312-HV3. Then Ffu312-HV3 was treated with EK to obtain HV3. Briefly, protein solutions were adjusted to pH 7.0–8.0, then the fusion protein Ffu312-HV3 was cleaved with EK (0.25%, w/w) at 23 °C overnight. After digestion and centrifugation, the supernatants were examined using Tricine/SDS-PAGE due to the low molecular weight of HV3. The cleaved proteins were then purified from the mixture of fusion tags and EK by nickel affinity chromatography according to the methods described above. The excretory proteins in the supernatant were quantified by Bradford assay. The collected fraction was analyzed by Tricine/SDS-PAGE. The thrombin neutralizing activity of Ffu312-HV3 and HV3 was expressed as anti-thrombin units (ATU). The anti-thrombin activity was measured quantitatively as follows: 0.2 mL of bovine fibrinogen (0.05%, w:v) in Tris–HCl buffer (pH 7.4) was incubated at 37 °C with 0.01–0.1 mL of the sample solution. Then, aliquots of 5 μL of thrombin standard solution (100 NIH unit mL^−1^) were added progressively at an interval of 1 min and mixed gently. The titration ended when a fibrin clot formed within 1 min [17]. The amount of sample that neutralizes one NIH unit of thrombin is one ATU.

## Additional file

**Additional file 1: Figure S1.** Expression levels and solubility of targeted proteins (RVs, CARDS TX, VEGFR-2 and Omp85) fused with His tag. Lane 1, 2 shows the soluble fraction and inclusion bodies of His-fused RVs; lane 3,4 shows the soluble fraction and inclusion bodies of His-fused CARDS TX; lane 5, 6 shows the soluble fraction and inclusion bodies of His-fused VEGFR-2; lane 7, 8 shows the soluble fraction and inclusion bodies of His-fused Omp85. Keys:Soluble fractions (S) and inclusion bodies (ib). The conditions for expression of the proteins were: 0.5 mM IPTG at 25 ℃. **Figure S2.** The purification of fusion protein Ffu217-CARDS TX after osmotic shock. Keys: M presents protein molecular weight marker; lane 1 presents the soluble fraction of fusion protein Ffu217-CARDS TX, lane 2 presents the final purified Ffu217-CARDS TX. **Figure S3.** (A) NanoLC-MS/MS analysis of protein Ffu217-CARDS TX by chymotrypsin digestion; (B) NanoLC-MS/MS analysis of protein Ffu217-CARDS TX by trypsin digestion. **Table S1.** The primers used in this study.

## Abbreviations

*E. coli*: *Escherichia coli*
*β*-FFase: *β*-fructofuranosidase
MBP: maltose-binding protein
NusA: N-utilization substance A
CARDS TX: *Mycoplasma pneumonia* community-acquired respiratory distress syndrome toxin
VEGFR-2: vascular endothelial growth factor receptors-2
RVs: rubella virus structural polyprotein
Omp85: chlamydia major outer membrane protein
HV3: hirudin variant III
EK: enterokinase sequence
