# Supplementary material for: A novel Ffu fusion system for secretory expression of heterologous proteins in Escherichia coli
Source: Microb Cell Fact. 2017 Dec 21;16:231. doi: 10.1186/s12934-017-0845-z (PMC5740907; doi:10.1186/s12934-017-0845-z)
Supplement: Supplementary file 1 — Additional file 1: Figure S1. Expression levels and solubility of targeted proteins (RVs, CARDS TX, VEGFR-2 and Omp85) fused with His tag. Lane 1, 2 shows the soluble fraction and inclusion bodies of His-fused RVs; lane 3,4 shows the soluble fraction and inclusion bodies of His-fused CARDS TX; lane 5, 6 shows the soluble fraction and inclusion bodies of His-fused VEGFR-2; lane 7, 8 shows the soluble fraction and inclusion bodies of His-fused Omp85. Keys:Soluble fractions (S) and inclusion bodies (ib). The conditions for expression of the proteins were: 0.5 mM IPTG at 25 ℃. Figure S2. The purification of fusion protein Ffu217-CARDS TX after osmotic shock. Keys: M presents protein molecular weight marker; lane 1 presents the soluble fraction of fusion protein Ffu217-CARDS TX, lane 2 presents the final purified Ffu217-CARDS TX. Figure S3. (A) NanoLC-MS/MS analysis of protein Ffu217-CARDS TX by chymotrypsin digestion; (B) NanoLC-MS/MS analysis of protein Ffu217-CARDS TX by trypsin digestion. Table S1. The primers used in this study. [file 12934_2017_845_MOESM1_ESM.docx]

**Additional Files:**

**Figure S1.** Expression levels and solubility of targeted proteins (RVs, CARDS TX, VEGFR-2 and Omp85) fused with His tag. Lane 1,2 shows the soluble fraction and inclusion bodies of His-fused RVs; lane 3,4 shows the soluble fraction and inclusion bodies of His-fused CARDS TX; lane 5,6 shows the soluble fraction and inclusion bodies of His-fused VEGFR-2; lane 7,8 shows the soluble fraction and inclusion bodies of His-fused Omp85. Keys：Soluble fractions (S) and inclusion bodies (ib).The conditions for expression of the proteins were: 0.5 mM IPTG at 25℃.


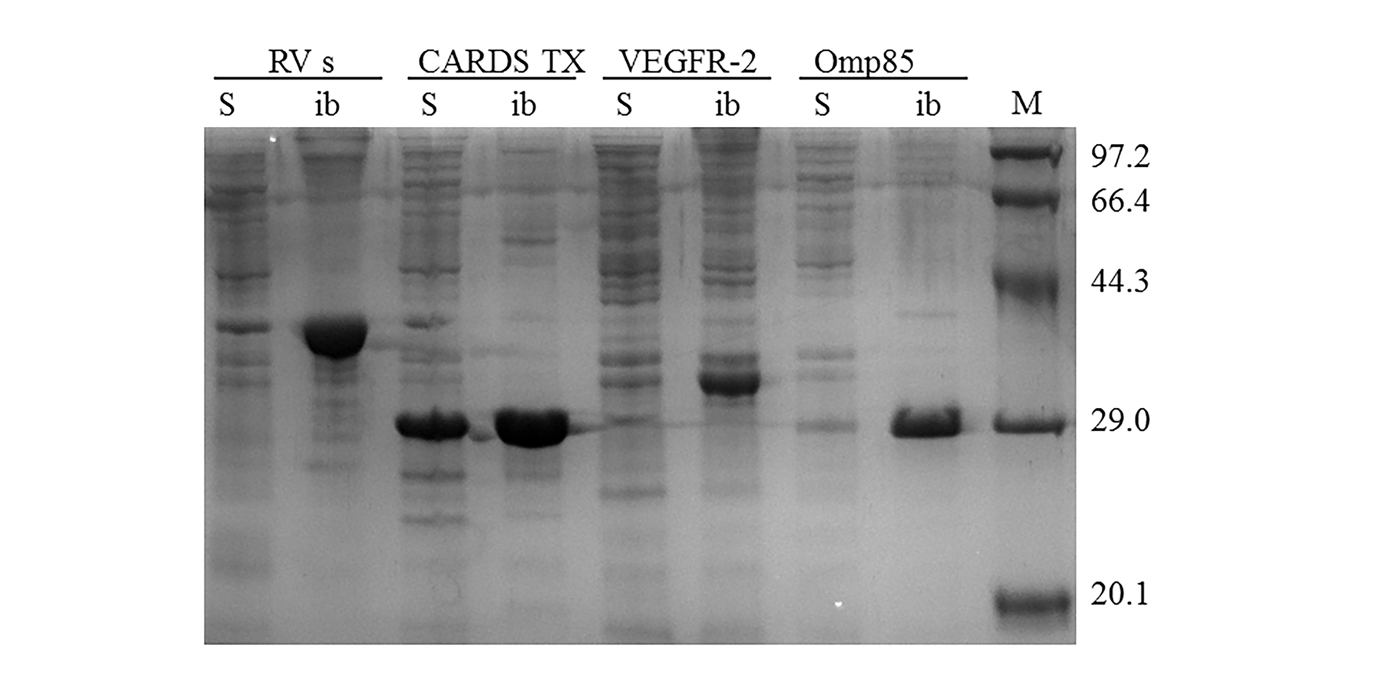


**Figure S2.** The purification of fusion protein Ffu217-CARDS TX after osmotic shock. Keys: M presents protein molecular weight marker; lane 1 presents the soluble fraction of fusion protein Ffu217-CARDS TX, lane 2 presents the final purified Ffu217-CARDS TX.


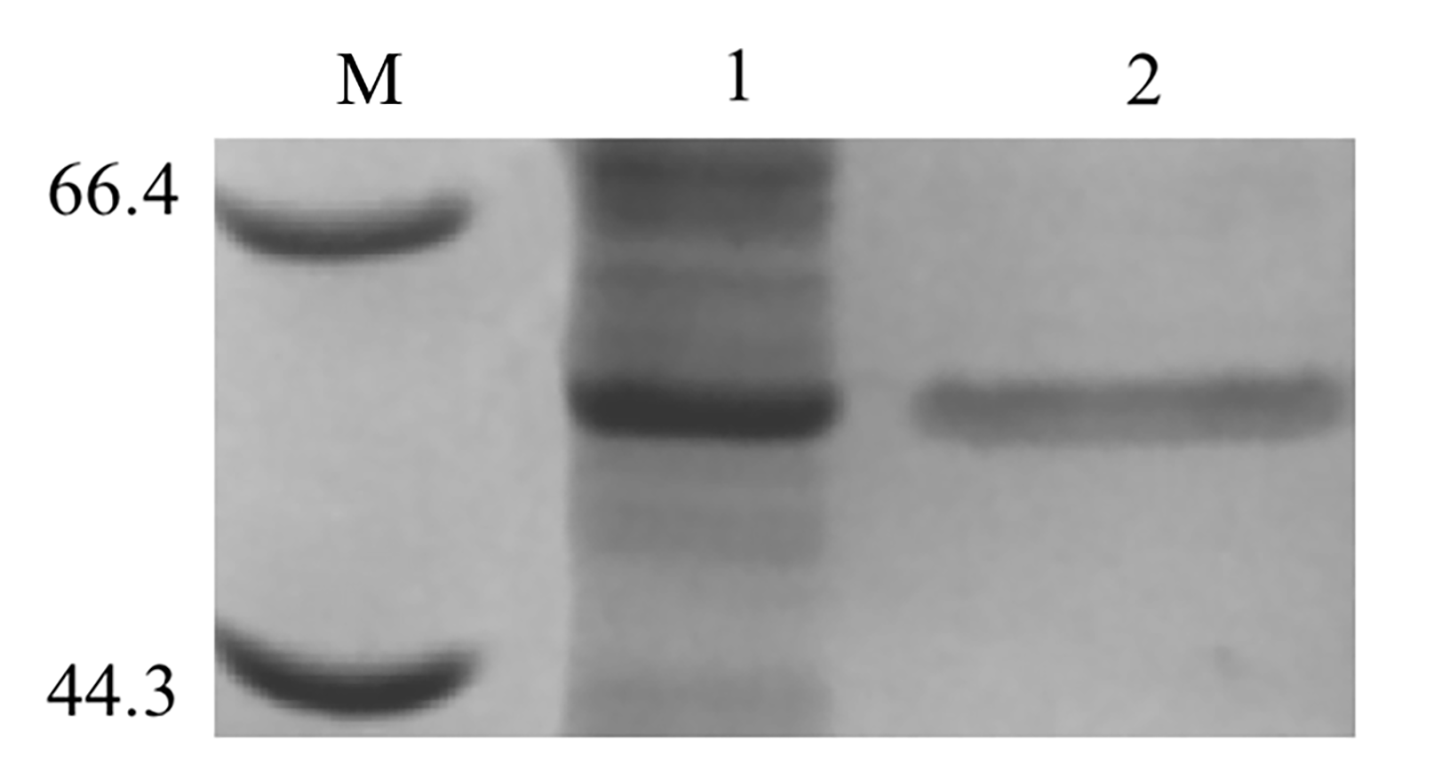


**Figure S3. (A)** NanoLC-MS/MS analysis of protein Ffu217-CARDS TX by chymotrypsin digestion;**(B)** NanoLC-MS/MS analysis of protein Ffu217-CARDS TX by trypsin digestion


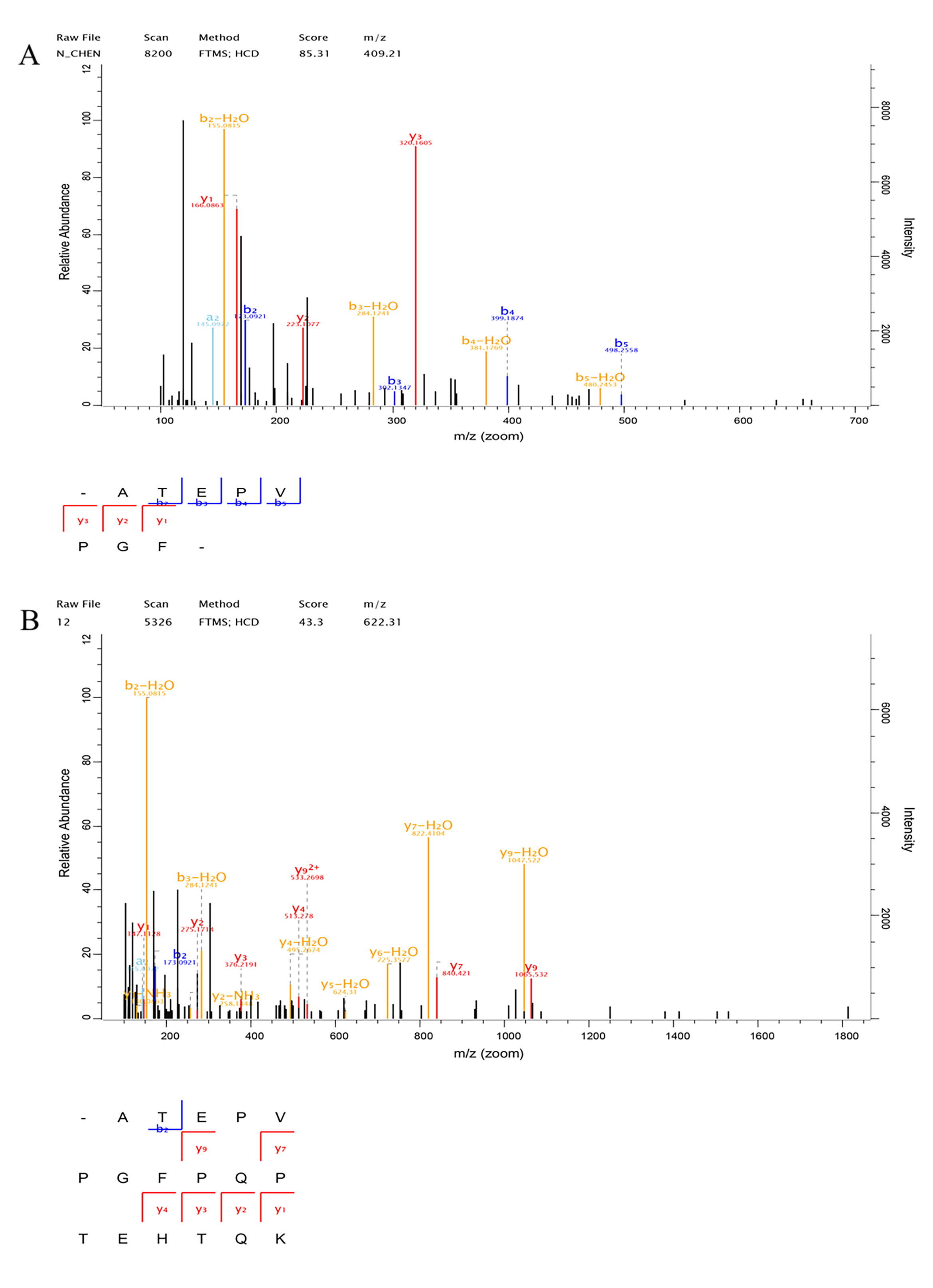


**Table S1:** The primers used in this study

| Primers | Use | Sequences（5’-3’） |
| --- | --- | --- |
| F1 | Upstream  for amplification of *bff* fragment | CATGCCATGGTGGAGAGAGCGTGTGTC |
| R2 | Downstream  for amplification of *bff* fragment | CCGGAATTC TTACTTTGCTACTGCTTTGCC |
| R3 | Downstream  for amplification of *bff_209_* fragment | GGGTTTCATATGCTTGTCATCGTCATCCTGGTCCTCGAAGATCTTG |
| R4 | Downstream  for amplification of *bff*_217_ fragment | GGGTTTCATATGCTTGTCATCGTCATCGGACCACTGAGTCTGGTG |
| R5 | Downstream  for amplification of *bff*_312_ fragment | GGGTTTCATATGCTTGTCATCGTCATCGACCTCATCCACGGTTTC |
| *hv3*-F | Upstream  for amplification of *hv3* fragment | ggaattcCATATGATGATCACCTACACTGACTG |
| *hv3*-R | Downstream  for amplification of *hv3* fragment | CCGCTCGAGTTACTATTCATCGTACGC |
| *Omp85*-Nde-F | Upstream  for amplification of *Omp85* fragment in Ffu fusion vector series | ggaattcCATATGCTGCGTGGCGGTGGCGAAC |
| *Omp85*-Xho-R | Downstream  for amplification of *Omp85* fragment in  Ffu fusion vector series | CCGCTCGAGTCAACCACCCTGCGGTTCGGTTG |
| *vegfr-2*-Nde-F | Upstream  for amplification of *Vegfr*f2 fragment in  Ffu fusion vector series | ggaattcCATATGAATACCACGCTGCAAATTAC |
| *vegf*r-2-Xho-R | Downstream  for amplification of *Vegfr2* fragment in Ffu fusion vector series | CCGCTCGAGTTAGGTAGAATTTTTCTTCG |
| *rvs*-Nde-F | Upstream  for amplification of *RVs* fragment in Ffu fusion vector series | ggaattcCATATGCCGGGTTGCGCTACTCAGAC |
| *rvs*-Xho-R | Downstream  for amplification of *RVs* fragment in Ffu fusion vector series | CCGCTCGAGCTTATTTACGAGCCTGGGAACCG |
| *cards tx*-Nde-F | Upstream  for amplification of *CARDS TX* fragment in Ffu fusion vector series | ggaattcCATATGCCGAACCCGGTCCGCTTTGTCTATC |
| *cards tx*-Xho-R | Downstream  for amplification of *CARDS TX* fragment in Ffu fusion vector series | CCGCTCGAGAGCGATACATTTGTCCAGCGGATTTTC |
